# Pediatric prognostic models predicting inhospital child mortality in resource‐limited settings: An external validation study

**DOI:** 10.1002/hsr2.1433

**Published:** 2023-08-27

**Authors:** Morris Ogero, John Ndiritu, Rachel Sarguta, Timothy Tuti, Samuel Akech

**Affiliations:** ^1^ Department of Mathematics University of Nairobi Nairobi Kenya; ^2^ Department of Infectious Disease Epidemiology London School of Hygiene & Tropical Medicine London United Kingdom; ^3^ Kenya Medical Research Institute (KEMRI)‐Wellcome Trust Research Programme Nairobi Kenya; ^4^ School of Medicine University of Nairobi Nairobi Kenya

**Keywords:** epidemiology, model external validation, pediatrics and adolescent medicine

## Abstract

**Background and Aims:**

Prognostic models provide evidence‐based predictions and estimates of future outcomes, facilitating decision‐making, patient care, and research. A few of these models have been externally validated, leading to uncertain reliability and generalizability. This study aims to externally validate four models to assess their transferability and usefulness in clinical practice. The models include the respiratory index of severity in children (RISC)‐Malawi model and three other models by Lowlavaar et al.

**Methods:**

The study used data from the Clinical Information Network (CIN) to validate the four models where the primary outcome was in‐hospital mortality. 163,329 patients met eligibility criteria. Missing data were imputed, and the logistic function was used to compute predicted risk of in‐hospital mortality. Models' discriminatory ability and calibration were determined using area under the curve (AUC), calibration slope, and intercept.

**Results:**

The RISC‐Malawi model had 50,669 pneumonia patients who met the eligibility criteria, of which the case‐fatality ratio was 4406 (8.7%). Its AUC was 0.77 (95% CI: 0.77−0.78), whereas the calibration slope was 1.04 (95% CI: 1.00 −1.06), and calibration intercept was 0.81 (95% CI: 0.77−0.84). Regarding the external validation of Lowlavaar et al. models, 10,782 eligible patients  were included, with an in‐hospital mortality rate of 5.3%. The primary model's AUC was 0.75 (95% CI: 0.72−0.77), the calibration slope was 0.78 (95% CI: 0.71−0.84), and the calibration intercept was 0.37 (95% CI: 0.28−0.46). All models markedly underestimated the risk of mortality.

**Conclusion:**

All externally validated models exhibited either underestimation or overestimation of the risk as judged from calibration statistics. Hence, applying these models with confidence in settings other than their original development context may not be advisable. Our findings strongly suggest the need for recalibrating these model to enhance their generalizability.

## 1 INTRODUCTION

Childhood mortality remains high in low and middle‐income countries (LMICs) despite a significant reduction since 1990, but it's uncertain how much in‐hospital mortality has changed over this duration.^1^, ^2^ Mortality rates among hospitalized children in sub‐Saharan Africa remain high, and most deaths occur within the first few hours of admission.^3^ An example of such a comparison can be found in Sharrow et al.^4^ which reported a marked disparity in under‐five mortality rates between low‐income and high‐income settings, with rates of 67% and 5%, respectively. Emergency Triage Assessment and Treatment (ETAT) guidelines, produced by the World Health Organization (WHO), guide immediate care for children admitted to hospitals. ETAT guidelines provide guidance on triage and use syndrome‐based approach to the management of common childhood conditions, but they are still used inconsistently and implemented suboptimally in Africa despite having been in existence since 2005.^5^

Regardless of an underlying condition, children at risk of death during hospitalization often present with similar danger signs and prompt triage and immediate supportive management are most important in reducing mortality and morbidity in admitted children.^6^ Identification of children at risk of in‐hospital mortality is the first step in directing supportive treatments that have the potential to reduce deaths. Therefore, clinical prediction models that identify the sickest children immediately upon arrival at the hospital for immediate supportive care and targeted close monitoring may be useful.^7^ However, many prognostic models do not meet methodological standards, reducing their utility and generalizability.^8^, ^9^ A common methodological weakness is the small sample size which makes a resultant model to have a low signal‐to‐noise ratio, limited number of events‐per‐variable (EPV), with some having EPV of less than 20, which is thought to lead to biased estimates.^10^, ^11^, ^12^, ^13^ Other weaknesses include poor handling of incomplete data, inappropriate statistical analyses, and optimistic interpretations of the model output. Furthermore, overreliance on fully automated statistical techniques, such as the stepwise model selection algorithm (backward or forward), which do not require expert or consensus input, can lead to overoptimistic or biased models that are not always relevant in routine practice.^11^, ^14^, ^15^, ^16^

Independent external validation of risk scores is recommended to assess their transferability and generalizability to other patient populations before their use in clinical practice. However, few models have been validated externally hence most models have uncertain reliability and generalizability.^15^, ^17^, ^18^ In this study, we conduct external validation of four prognostic models predicting in‐hospital mortality for pediatric patients in LMICs using routine hospital data collected by the Clinical Information Network (CIN) in Kenya. These models were identified in the recent systematic review of pediatric prognostic models.^8^, ^9^

## 2 METHODS

### 2.1 Ethics and reporting

This external validation study followed recommendations for developing, reporting and validating prognostic studies stipulated in the TRIPOD (Transparent Reporting of a multivariable prediction model for Individual Prognosis or Diagnosis) statement.^12^, ^13^ Kenya Medical Research Institute's (KEMRI) scientific and ethical review committee approved the CIN study (#3459). The Kenya Ministry of Health (MoH) permitted this work using deidentified routine patient care data abstracted from medical records after discharge without individual patient/clinician consent.

### 2.2 Patient and public involvement

Patients were not involved in the study's design, so research questions were not informed by patients' priorities, experiences, or preferences.

### 2.3 Study design and setting

**Figure 1:**
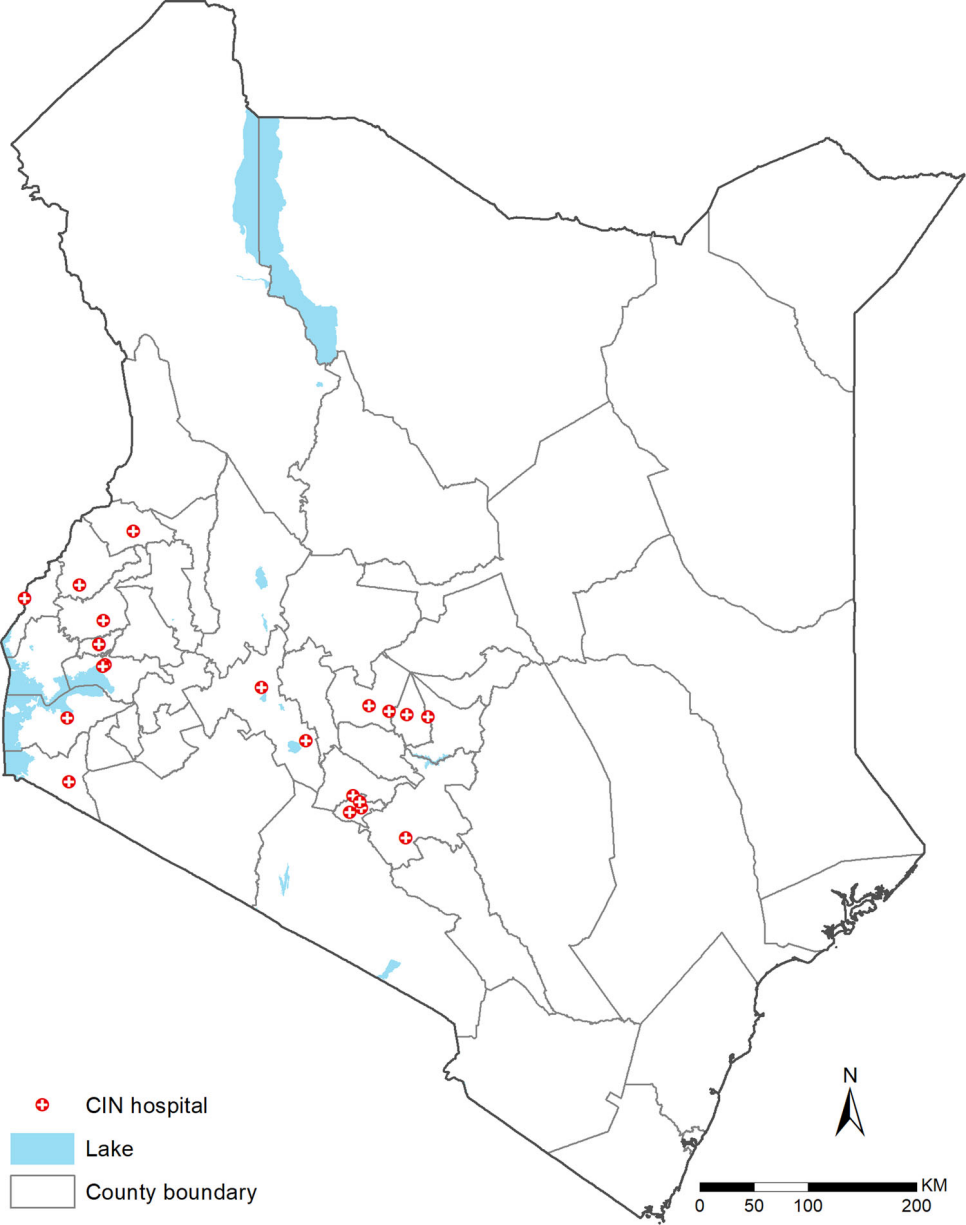
Locations of hospitals included in the validation cohort. CIN, Clinical Information Network.

This validation study uses data collected from CIN, which comprises 20 public county referral (previously district) hospitals in Kenya whose locations are shown in Figure 1. These hospitals serve as first‐referral level hospitals but also serve patients who seek care directly from their homes. The CIN is a collaboration between researchers from the KEMRI‐Wellcome Trust Research Programme (KWTRP), the Kenya MoH, the Kenya Paediatric Association, and participating hospitals. The selection of these hospitals under CIN was purposeful to represent wide geographical variation in Kenya and targeted those with a high volume of pediatric admissions, at least 1000 children per year. More details about the selection of these hospitals and their locations have been given elsewhere.^3^

### 2.4 Outcome

In our study, the outcome of interest was all‐cause in‐hospital pediatric mortality. To minimize bias in outcome assessment, the hospital data clerks who abstracted data from medical records were kept unaware of the study, ensuring their inability to influence the outcome assessment and maintaining blindness to predictors. We aimed to comprehensively represent the target population by considering all pediatric patients admitted to the hospital during the study period. By including all eligible patients, we sought to avoid systematic selection bias related to the outcome of interest.

### 2.5 Prognostic models included for external validation

Models included in this external validation were obtained from a recent systematic review of prognostic models,^9^ which identified 11 models predicting in‐hospital mortality for children admitted in LMICs hospitals. However, we excluded nine models^19^, ^20^, ^21^, ^22^, ^23^, ^24^ since they did not publish the relative weights of the risk factors and model intercept for the logistic regression models or the baseline hazard function for the survival models as required for the external validation. Before we decided to exclude these models, we contacted the corresponding authors of those studies by email, asking for the complete model formula without success. The following are the models included in the current external validation study.

#### 2.5.1 Respiratory index of severity in children (RISC)‐Malawi prognostic model

RISC‐Malawi is a predictive model developed using prospectively collected clinical data from a cohort of 14,665 hospitalized children aged 2−59 months with pneumonia diagnosis in Malawi between 2011 and 2014. The total number of deaths in the model development cohort was 465, and the case‐fatality rate was 3.2% across the seven hospitals under study.^25^ The authors utilized logistic regression to develop a prognostic model whose intercept and odds ratios for the seven prognostic factors are provided in Table 1. The author reported an area under the receiver operator characteristic curve (ROC) of 0.79 (95% CI: 0.76−0.82), demonstrating a fair ability to discriminate children's mortality risk.

**Table 1:** Models to be externally validated.

Study	Models	Inclusion criteria	Predictors	Model equation with intercept and odds ratios	Model derivation AUC (95% CI)	CIN sample size eligible for validation (% with mortality outcome)
Hooli et al.^25^	RISC‐Malawi model	Age ≥2 and ≤59 months, pneumonia by danger signs (cough or difficult breathing and at least one danger sign (central cyanosis, grunting, chest wall indrawing, stridor, inability to drink, AVPU<A, or convulsion))	Moderate hypoxemia, severe hypoxemia, moderately malnourished, severely malnourished, child‐sex (female), wheezing	=−4.67+(moderate hypoxemia×0.43)+(severe hypoxemia×1.62)+(moderately malnourished×0.55)+(severely malnourished×1.53)+(femalesex×0.22)+(wheeze×−0.35)+(unconsciousness×1.74)	0.79 (95% CI: 0.76−0.82)	*N* = 50,669, mortality = 4406 (8.7%)
Lowlaavar et al.^26^	Primary model	Age ≥6 and ≤60 months with proven or suspected infection	Abnormal Blantyre coma score, positive HIV, weight for age *z*‐score	=−4.280+(abnormal Blantyre Coma Scale×2.51)+(Positive HIV×1.32)+weight for age z‐score×−0.2	0.85 (95% CI: 0.80−0.89)	*N* = 86,784, mortality = 4045 (4.7%)
	Model 2	Age ≥6 and ≤60 months with proven or suspected infection	Abnormal Blantyre coma score, positive HIV, middle upper arm circumference (MUAC)	=−0.523+(abnormalBlantyrecomascale×2.54)+(PositiveHIV×2.27)+(MUAC×−0.03)	0.84 (95% CI: 0.79−0.89)	*N* = 86,784, mortality = 4045 (4.7%)
	Model 3	Age ≥6 and ≤60 months with proven or suspected infection	Abnormal Blantyre coma score, MUAC	=0.303+(abnormalBlantyrecomascale×2.47)+(MUAC×−0.03)	0.82 (0.72‐0.91)	*N* = 86,784, mortality = 4045 (4.7%)

Abbreviations: AUC, area under the curve; CIN, Clinical Information Network; RISC, respiratory index of severity in children; WAZ, weight for age z‐score.

#### 2.5.2 Lowlaavar et al. prognostic models

Lowlaavar et al.^26^ developed three models utilizing a two‐site prospective observational study in Uganda which enrolled children between 6 months and 5 years admitted with a proven or suspected infection. In their study, 1307 children were enrolled consecutively, and 65 (5%) of participants died during their hospital stay. The study was conducted between March 2012 and December 2013. The primary model included three predictors namely weight‐for‐age *z*‐score (WAZ), Blantyre coma scale, and HIV status. Based on the derivation dataset, the area under the curve (AUC) was 0.85 (95% CI: 0.80−0.89). The second model included MUAC (mid‐upper arm circumference), Blantyre coma scale, and HIV status. The area under the ROC curve of this model was 0.84 (95% CI: 0.79−0.89). The third model included two variables MUAC and Blantyre coma scale, with an AUC of 0.82 (0.72−0.91). The equations of these three models are provided in Table 1.

### 2.6 Data collection

Patients admitted to CIN hospitals have the following data routinely collected and documented: biodata (e.g., age, gender), history of illness (e.g., length of illness, history of fever, diarrhea, vomiting, convulsions, vaccinations), examination findings (for instance vital signs, presence of thrush, edema, and visible wasting), investigations done during admission (e.g., malaria, hematology, glucose, HIV, and lumber puncture tests results), admission and discharge diagnoses, treatment received at admission (e.g., antibiotics, anti‐malarial, anti‐tuberculous medications), supportive care (oxygen support, blood transfusion, fluids, bolus fluids treatment), vital signs measurements for the initial 48 h of admission, and outcome at discharge. These patient details are systematically documented by clinicians and nurses using a standardized medical record form called Pediatric Admission Record (PAR),^27^ adopted for use by hospitals participating in CIN. Upon discharge or death of a patient, a trained data clerk abstracts these data to a customized data capture tool designed using a nonproprietary Research Electronic Data Capture (REDCap) platform.^28^ As part of quality assurance protocols, data quality checks (data completeness and transcription errors) are run locally using a script written in R programming language before data are synchronized to a central database. If any inconsistencies, data omissions or transcription errors occur, the data clerk corrects these after verification from the patient record. The data clerk does not make corrections to documentation errors made by the clinical or nursing teams.

### 2.7 Patient inclusion criteria

Patients hospitalized in pediatric wards across 20 CIN hospitals were eligible for inclusion from September 2013 to December 2021. Surgical cases of burns patients, healthy children accompanying sick babies, children admitted with poisonings such as organophosphate ingestion, or any other form of poisoning, and traumatic & road traffic cases were all excluded from the validation cohort. We also excluded patients admitted during healthcare workers (nurses and doctors) strikes.^29^ These exclusions were done to make the validation dataset as similar as possible to the derivation cohort of the models whose performance is assessed in this study. The following eligibility criteria were applied to obtain a model‐specific cohort for external validation.

#### 2.7.1 Eligibility criteria for RISC‐Malawi model's external validation cohort

**Figure 2:**
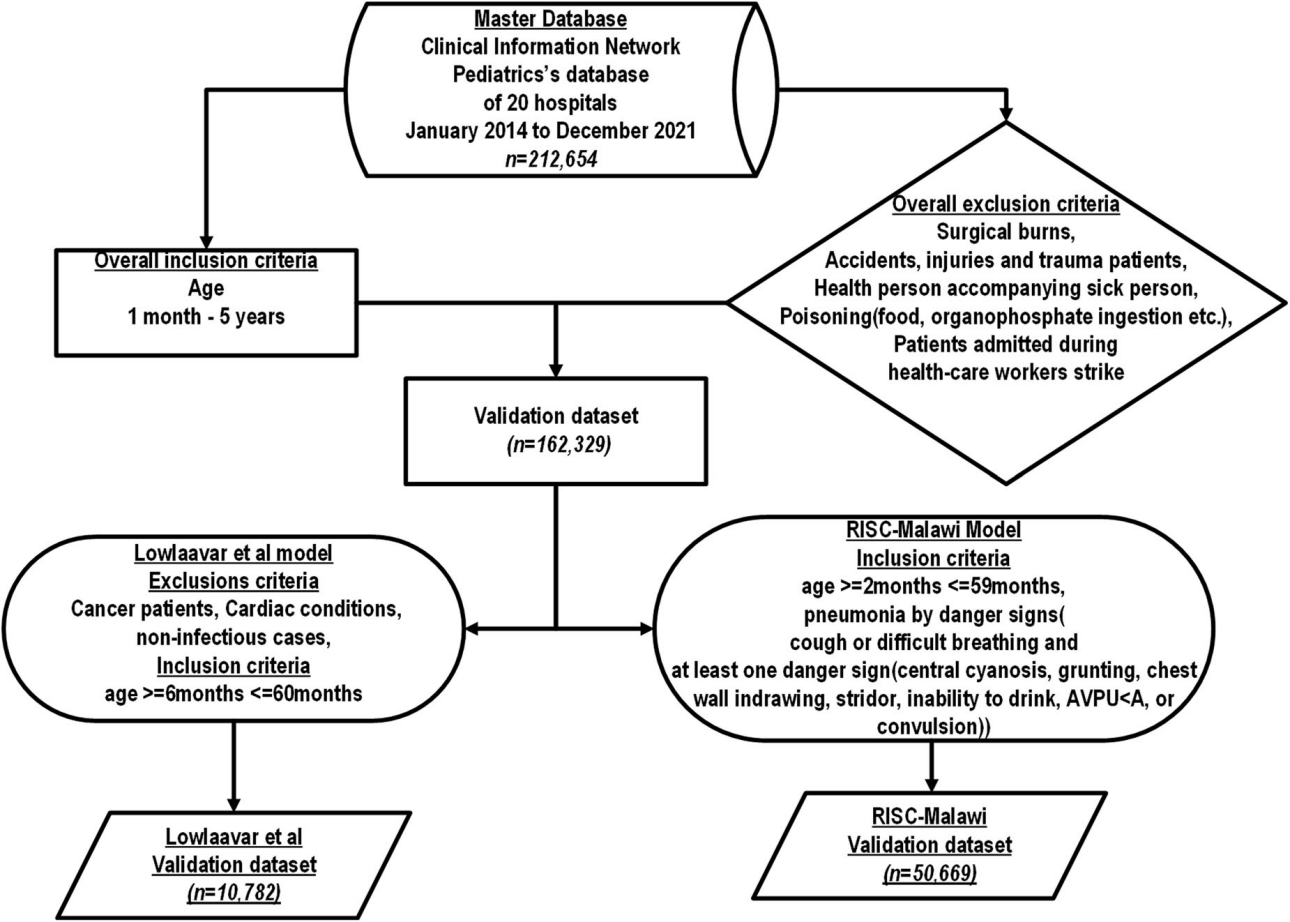
Flow chart of patients included in a model external validation cohort.

As defined in the study that derived RISC‐Malawi model, the external validation cohort included patients aged 2−59 months with admission diagnoses of pneumonia that were defined as follows; history of cough or difficulty breathing and at least one of the danger signs, which included central cyanosis, grunting, chest‐wall indrawing, stridor, inability to drink/breastfeed, and or painful responsive (P) or unresponsiveness (U) based on the disability scale of AVPU (Alert, Verbal, Painful responsive, unresponsive) see Figure 2.

RISC‐Malawi model predictors were defined in the validation cohort as follows; moderate malnourished was defined as MUAC between 11.5 cm and 13.5 cm. Severe malnourished was defined as MUAC < 11.5 cm. Unconsciousness was assessed using the disability scale of AVPU (Alert, Verbal, Pain, Unresponsive). Thus, patients were assumed to be unconscious if they responded to pain only or were unresponsive altogether (P or U). Moderate hypoxemia was defined as oxygen saturation ranging from ≥90% to ≤92%, and severe hypoxemia was defined as oxygen saturation <90%.

To understand the performance of the RISC‐Malawi model in a scenario where the definition of predictors varied from the original study, we performed sensitivity analyses where pneumonia diagnosis was defined based on the clinical diagnosis instead of danger signs.

#### 2.7.2 Eligibility criteria for Lowlavaar et al. models' external validation cohort

To match the clinical characteristics of the external validation cohort to that of the model derivation, we included children aged 6 to ≤60 months and excluded patients with the following features: malnourished cases (defined as clinical diagnoses of malnutrition), readmission cases, those with a cancer diagnosis, those with a heart condition, and patients with any parasitological confirmed or clinically suspected noninfectious illness see Figure 2.

Model predictors were defined as follows; weight‐for‐age *z*‐score was computed based on the reference materials in the WHO website (for patients <24 months)^30^, ^31^ and the National Centre for Health Statistics (for patients >24 months),^32^ and abnormal Blantyre coma score (BCS).

**Figure 3:**
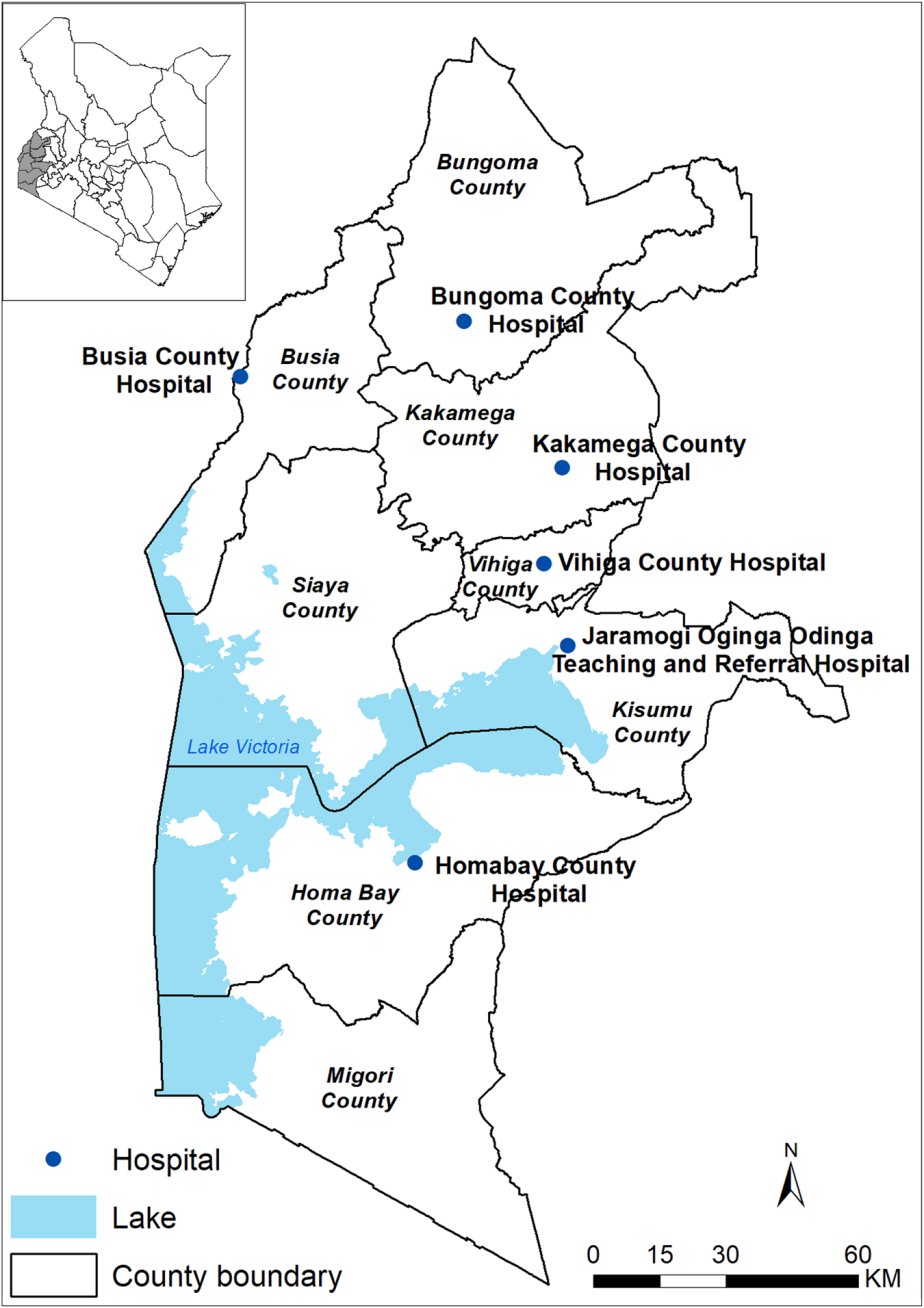
Locations of the six hospitals whose patients were included in the validation cohort of Lowlaavar et al. models.

Since BCS data was only collected in 6 out of 20 CIN hospitals, we limited the validation data to include patients from the 6 hospitals whose locations are shown in Figure 3. The collection of BCS data was introduced in September 2019 in the 6 hospitals participating in the WHO‐led study evaluating the subnational pilot introduction of the RTS,S/AS01 malaria vaccine in western Kenya—a region with high malaria transmission throughout the year.^33^ Therefore, as defined in the model derivation study, a patient with a BCS of less than five was considered abnormal.

### 2.8 Assessing performance of the prognostic model in the external validation

Model regression coefficients were used to determine predictions of the risk of mortality in the validation dataset as follows; for each patient in the validation cohort, the presence of the model predictor was assigned a value of 1, while its absence was assigned a value of 0. This was then multiplied by the corresponding regression coefficients and added together with the model intercept to get a linear predictor. The patient's predicted risk of in‐hospital mortality was then computed on the resultant linear predictor using the logistic function $\frac{exp(\beta_{0}+\beta_{1}X_{1}\;+\;\ldots {\;\beta }_{k}X_{k}}{1+exp(\beta_{0}+\beta_{1}X_{1}\;+\;\ldots {\;\beta }_{k}X_{k}}$, where $\beta_{0}$ is the model intercept and $\beta_{i}$ is the regression coefficient for a given predictor X.

Model performance was determined based on two metrics: discriminatory index and model calibration. To determine the discriminative ability of the model, we used the AUC, also known as c‐statistic which is a measure of the power of a model/score to distinguish between two classes.^11^ We classified the model's discriminatory ability using the following cutoffs; (AUC) ≥ 0.90 was classified as “excellent discrimination,” AUC ranging from 0.80 to 0.89 was classified as “good discrimination,” AUC ranging from 0.70 to 0.79 as “fair discrimination,” and “poor discrimination” was for the model whose AUC was <0.70.^34^, ^35^

Model calibration was assessed by plotting the predicted probability of in‐hospital death against the observed proportion. The observed proportion is calculated using the proportion of events that occurred relative to the total number of observations in the datasets used for validation. The predictive model calculates the probability of an event occurring for each observation in the dataset to obtain the predicted probability. Two calibration metrics are computed, namely calibration slope and calibration intercept.^36^ The calibration slope, which has a reference value of 1, examines the dispersion of the predicted risks such that a slope value of <1 suggests that estimated risks are too extreme, while a slope value of >1 indicates that the estimated risks are too low. On the other hand, the calibration intercept is a measure of calibration‐in‐the‐large and has a reference value of 0 such that a calibration intercept of <0 indicate overestimation while that of >0 indicates an underestimation of risk.^36^ The confidence intervals for both c‐statistic, calibration slope and intercept were calculated through bootstrap resampling using the CalibrationCurves package in R.^37^

### 2.9 Handling missing data

The two models selected for external validation in the current study used variables that had varying levels of documentation in the validation cohort. For instance, the data in MUAC, which was used to determine malnutrition status, were missing in 49.8% of the eligible population for the RISC‐Malawi model. Documentation of this variable was also not very well documented in the derivation dataset by Hooli et al. whereby the data were missing in 45.8% of the eligible population. See Table 4 for the data missingness of predictors in the RISC‐Malawi model and Table 5 for the Lowlavaal et al. models.

**Table 2:** Demographic and clinical characteristics of the cohort used to externally validate RISC‐Malawi model.

	All patients	Survived	Died
Population	*n* = 50,669	46,263/50,669 (91.3%)	4406/50,669 (8.7%)
Child‐sex (female)	22,184/50,669 (43.8%)	20,001/22,184 (90.2%)	2183/22,184 (9.8%)
Age (months) median (IQR)	13 (7−24)	14 (7−25)	9 (6−16)
Moderate hypoxemia	3875/50,669 (7.6%)	3591/3875 (92.7%)	284/3875 (7.3%)
Severe hypoxemia*	8949/50,669 (17.7%)	7696/8949 (86%)	1253/8949 (14%)
Moderately malnourished*	8699/50,669 (17.2%)	7988/8699 (91.8%)	711/8699 (8.2%)
Severely malnourished*	3042/50,669 (6%)	2438/3042 (80.1%)	604/3042 (19.9%)
Wheeze present	6666/50,669 (13.2%)	6181/6666 (92.7%)	485/6666 (7.3%)
Unconsciousness*	3221/50,669 (6.4%)	1940/3221 (60.2%)	1281/3221 (39.8%)

*Note*: Unconscious* defined as either painful responsive or unresponsive in the disability scale of AVPU (Alert, Verbal, Painful responsive, unresponsive). Severe hypoxemia* defined as oxygen saturation <90%. Severely malnourished* defined as mid‐upper arm circumference (MUAC) <11.5 cm. Moderately malnourished* defined as MUAC between 11.5 and 13.5 cm.

Abbreviation: RISC, respiratory index of severity in children.

To avoid bias that may have resulted from excluding observations with missing data, we undertook multiple imputations to account for the uncertainty caused by missing data.^38^, ^39^ To do this, we created 50 imputation datasets under the assumption of missing at‐random mechanism. Variables to be imputed were ordered based on their levels of data missingness from low to high. This was meant to fully benefit from the imputing algorithm's chained equations and boost convergence. The simulation error in the multiple imputations was minimized by using 100 iterations between imputations. Validation of the prognostic model was carried out on each of the imputed datasets. Rubin's rules^40^ were used to pool estimates from the 50 multiply‐imputed datasets. As recommended,^41^ a plot of densities of both the observed and imputed values suggested that the imputations generated from the imputing algorithm were plausible, as shown in Supporting Information: Figure 1.

All analyses were done using R 3.6.3 (R Foundation for Statistical Computing; http://www.cran.r-project.org).

## 3 RESULTS

### 3.1 Eligible population

The CIN's database had 212,654 patients admitted and 162,329 patients were eligible to be included in the validation cohort from all hospitals (*n* = 20). We further applied model‐specific exclusions, as shown in Figure 2 to obtain *n* = 50,669 and *n* = 10,782 patients eligible for the external validation of the RISC‐Malawi and Lowlaavar models, respectively. Model performance results are presented using multiply‐imputed datasets.

#### 3.1.1 Characteristics of the cohort used in the external validation of the RISC‐Malawi prognostic model

We had *n* = 50,669 patients who met the eligibility criteria to be included in the validation dataset of the RISC‐Malawi model. Out of this cohort, the pneumonia case fatality ratio was 8.7%, ranging from 1.9% to 16.3% across hospitals. Upon examining the characteristics of this cohort, we observed that 3221/50,669 (6.4%) of the patients were unconscious, of which 1281/3221 (39.8%) died. 3042/50,669 (6%) of all patients were severely malnourished, and 604 (19.9%) of them died. In addition, the data also suggested 8949/50,669 (17.7%) patients experienced severe hypoxemia, of which 14% (1253/8949) died, as shown in Table 2.

#### 3.1.2 Characteristics of the cohort used in the external validation of the Lowlaavar et al. models

Since the derivation study of the Lowlaavar models included the BCS as a model predictor, the eligibility criteria for the model validation cohort included patients from six hospitals where the BCS data was collected. In this dataset, 10,782 children met the eligibility criteria, of which 570/10,782 (5.3%) experienced in‐hospital mortality. As defined in the model's derivation study, patients with a BCS <5 were considered to have abnormal BCS that was present in 1199 patients, of whom 236 (19.7%) died in the hospital, as shown in Table 3.

**Table 3:** Demographic and clinical characteristics patients from six hospitals who were included in the validation cohort of Lowlaavar et al. models.

Indicator	All patients	Survived	Died
Population	*N* = 10,782	10,212/10,782 (94.7%)	570/10,782 (5.3%)
Gender (female)	4508/10,782 (41.8%)	4245/4508 (94.2%)	263/4508 (5.8%)
Age (months) median (IQR)	24 (14−42)	24 (14−42)	22.5 (11−38)
HIV diagnosis	75/10,782 (0.7%)	63/75 (84%)	12/75 (16%)
Abnormal BCS	1199/10,782 (11.1%)	963/1199 (80.3%)	236/1199 (19.7%)
WAZ	−0.5 (−1.5 to 0)	−0.5 (−1.5 to 0)	−1 (−2 to 0)
MUAC	14.3 (13.5−15)	14.3 (13.6−15)	14 (13.1−14.8)

*Note*: Abnormal BCS defined as BSC of <5.

Abbreviations: BSC, Blantyre coma score; MUAC, mid‐upper arm circumference; WAZ, weight for age *Z*‐score.

#### 3.1.3 Comparison of patients' profile between validation and derivation cohort

A comparative analysis of patients' case mix between the validation and derivation cohort of the RISC‐Malawi model indicates a satisfactory degree of similarity in their characteristics. However, it is noteworthy that the dataset employed to validate the RISC‐Malawi model exhibited a relatively higher pneumonia case fatality rate (8.7%) compared to the dataset used in its derivation (3.2%). Additionally, there was a significantly higher prevalence of unconsciousness in the validation dataset (6.4%) compared to the derivation cohort (3.7%), as shown in Table 4. Similarly, the dataset employed in developing the Lowlaavar models demonstrated a mortality rate of 5% which was comparable to the mortality rate of the dataset used for validation purposes (5.3%). However, the derivation cohort exhibited relatively higher levels of malnutrition, as evidenced by their MUAC values and stunting levels, as presented in Table 5.

**Table 4:** Comparing predictors used in RISC‐Malawi model in derivation and validation datasets.

Predictor	Variable in model derivation dataset	Variable equivalent in external validation dataset	*N* in the derivation dataset	*N* in the validation dataset
Oxygen saturation	Normal	Oxygen saturation 93%−100%	10,586 (64.3%)	16,897 (33.3%)
	Moderate hypoxemia (90%−92%)	Oxygen saturation 90%−92%	1382 (8.4%)	3875 (7.6%)
	Severe hypoxemia	Oxygen saturation <90%	2094 (12.7%)	8949 (17.7%)
	Missing oxygen saturation		2413 (14.7%)	20,947 (41.3%)
Malnutrition	Normal	MUAC >13.5 cm	4557 (27.7%)	15,234 (30.1%)
	Moderately malnourished	MUAC (11.5−13.5 cm)	3382 (20.5%)	8699 (17.2%)
	Severely malnourished	MUAC <11.5 cm	991 (6.0%)	3042 (6.0%)
	Missing MUAC data		7545 (45.8%)	25,232 (49.8%)
Wheeze	Wheezing = yes	Wheezing = yes	4117 (25.0%)	6666 (13.2%)
	Wheezing = no	Wheezing = no	8767 (53.2%)	42,701 (84.3%)
	Missing wheezing data	Missing data	3591 (21.8%)	1302 (2.6%)
Unconsciousness	Unconscious = yes	Painful responsive or unresponsive in the disability scale of AVPU (Alert, Verbal, Painful responsive, unresponsive)	608 (3.7%)	3221 (6.4%)
	Unconscious = no	Alert or verbal response based on the disability scale of AVPU	12,529 (76.1%)	45,915 (90.6)
	Missing data		3338 (20.3%)	1533 (3.0%)

Abbreviations: MUAC, mid‐upper arm circumference; RISC, respiratory index of severity in children.

**Table 5:** Comparing predictors used in Lowlavaal et al. model in derivation and validation datasets.

Predictor	Variable in model derivation dataset	Variable equivalent in external validation dataset	*N* in the derivation dataset	*N* in the validation dataset
Blantyre coma score	Abnormal Blantyre coma score (score <5)	Verbal response based on the disability scale of AVPU	Not provided	2023 (2.3%)
		Missing data	Not provided	103 (1.0%)
HIV diagnosis	Positive HIV diagnosis	Positive HIV diagnosis	66 (5.1%)	850 (1.0%)
Weight for age *z*‐score (WAZ)	Severely stunted (WAZ < −3)	Weight for age *z*‐score < −3	206 (15.9%)	481 (4.5%)
	Underweight (WAZ < −2)	Weight for age *z*‐score < −2	372 (28.6%)	2649 (24.7%)
	Missing data		No provided	45 (0.4%)
Mid‐upper arm circumference	MUAC < 125 mm	MUAC < 125 mm	187 (14.5%)	898 (8.8%)
	MUAC < 115 mm	MUAC < 115 mm	94 (7.3%)	292 (2.8%)
	Missing data		No provided	531 (4.9%)

Abbreviation: MUAC, mid‐upper arm circumference.

#### 3.1.4 Performance of RISC‐Malawi prognostic model in external validation dataset

**Figure 4:**
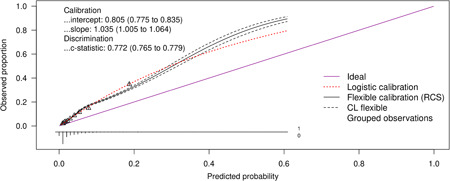
Performance of the RISC‐Malawi model in an external validation dataset. The figures show calibration curves and other model performance metrics. CL denotes confidence limits (95%); RCS denotes restricted cubic splines.

The discriminatory ability (c‐statistic) of the RISC‐Malawi model was 0.77 (95% CI: 0.77−0.78), whereas the calibration slope was 1.04 (95% CI: 1.00−1.06). The calibration intercept was 0.81 (95% CI: 0.77−0.84), indicative of a poorly calibrated model since it underestimates the risk (intercept >0) see Figure 4.

#### 3.1.5 Performance of Lowlaavar et al. models in external validation dataset

**Figure 5:**
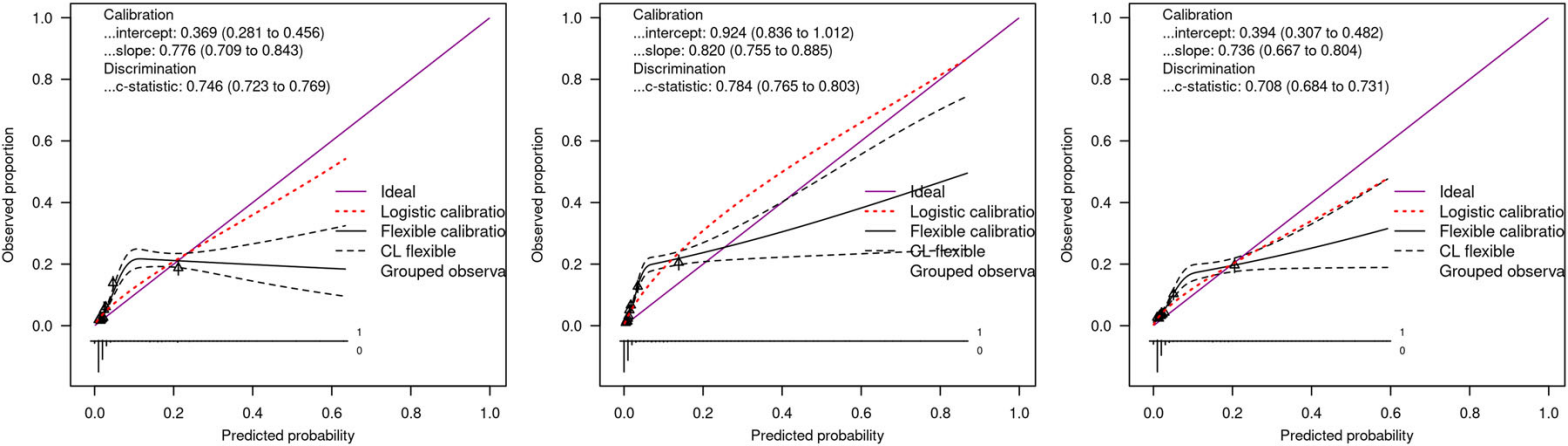
Performance of the Lowlaavar et al. (2016) models in an external validation dataset where abnormal Blantyre coma score (BCS) was defined as BCS < 5. The first panel to the left is the calibration curves of the primary model (model I), the panel in the middle are the calibration curves for model II, and the last panel to the right are the calibration curves of model III. CL denotes confidence limits (95%); RCS denotes restricted cubic splines.

We computed the performance statistics for the three models derived by Lowlaavar et al. and the findings were as follows; the primary model (model 1), which included three predictors (abnormal BCS, HIV+, weight for age *z*‐score) had a c‐statistic of 0.75 (95% CI: 0.72−0.77) while the calibration slope was 0.78 (95% CI: 0.71−0.84), and the calibration intercept was 0.37 (95% CI: 0.28−0.46). The second model (model 2) included the following three predictors: abnormal BCS, HIV+, and MUAC had a c‐statistic of 0.78 (95% CI: 0.77−0.80) while the calibration slope was 0.82 (95% CI: 0.76−0.89) and the calibration intercept was 0.92 (95% CI: 0.84−1.10). The third model with two predictors (abnormal BCS and MUAC) had a c‐statistic of 0.71 (95% CI: 0.68−0.73), while the calibration slope was 0.73 (95% CI: 0.67−0.80), and the calibration intercept was 0.39 (95% CI: 0.31−0.48) as shown in Figure 5.

### 3.2 Sensitivity analyses

As a sensitivity analysis, we varied the criteria of determining pneumonia diagnosis in the validation cohort of RISC‐Malawi such that instead of using danger signs (central cyanosis, grunting, indrawing, stridor, inability to drink, AVPU, and convulsion) as used in the original study to define pneumonia, we used clinical admission diagnosis of pneumonia. All other eligibility criteria remained unchanged, resulting in a sample size of 56,045 with a pneumonia case fatality rate of 7.6%. Evaluation of the RISC‐Malawi model performance in the sensitivity analyses dataset suggested a reduced performance compared to what was seen in the primary analyses, as shown in the Supporting Information: Figure 2.

We also undertook a sensitivity analysis of the Lowlavaar models using patients from all hospitals (*n* = 20) instead of the six hospitals as used in the main analyses. However, the abnormal BCS was defined using AVPU scores which is a disability scale such that patients who were at “V” during the clinical assessment by a physician were classified as having abnormal Blantyre scale. Those who met the eligibility criteria were 86,784 patients and in‐hospital mortality was 4.7% (*n* = 4045). Patients with abnormal BCS were 2023 (2.3%) out of which 268 (13.5%) died. Performance of the Lowlavaar models in the sensitivity analyses dataset were lower as compared to the main analyses as shown in the Supporting Information: Figure 3.

## 4 DISCUSSION

### 4.1 Summary of findings

The first step towards wider clinical application of clinical prediction rules is to validate existing prognostic models to identify children at risk of deterioration in diverse settings. In this study, we externally validated four predictive models initially developed by two studies to identify children at an increased risk of in‐hospital mortality in low‐resource settings.^25^, ^26^

Using a diverse population of children admitted to 20 hospitals from 2014 to December 2021, we performed an external validation to assess these four prognostic scores' discriminatory ability and calibration levels. All models had fair discriminatory values (AUC: 0.70−0.79). However, they all underestimated the mortality (calibration intercept >0), leading to the misclassification of patients at an increased risk of deterioration. These model performance measures were even lower when they were validated using the sensitivity analyses datasets where we varied the definitions of abnormal BCS and pneumonia from how it was defined in the original study by Lowlavaar et al. and Hooli et al. for RISC‐Malawi, respectively. This demonstrates the value addition of explaining predictors as used in the model derivation study.

The suboptimal performance of these models in the CIN data sets may be due to having more diverse patient populations and different case‐mix. Although we attempted to ensure that the patients' characteristics in the validation and derivation cohort were as similar as possible, we observed that the cohort we used to validate the RISC‐Malawi model had a higher pneumonia case fatality rate (8.7%) than the original patient group (3.2%). Despite this difference, the discriminatory ability of the RISC‐Malawi in the validation cohort had an AUC 0.77 (95% CI: 0.77−0.78), which was nearly similar to that observed in the model derivation cohort 0.79 (95% CI: 0.76−0.82).

While the calibration intercepts of all models we externally validated suggested underestimation of the risk of mortality in their predictions, calibration slopes of the same models illustrated that these predictions were too extreme, especially for the Lowlaavar et al. models whose calibration slopes were all <1. On the other hand, predictions of the risk of pneumonia‐related mortality by the RISC‐Malawi model were too low, as judged by the calibration slope of >1. The result may be partly explained by the inclusion of more physical examination variables as prognostic factors, which could make a model underperform in an external dataset because of the variations in interobserver agreement, which is more common in physical examination findings.^42^, ^43^ It is encouraged to include prognostic factors that do not have interobserver variations, such as blood lactate and other biomarkers, including C‐reactive protein, procalcitonin, and so forth, in the settings where these tests are available. However, while such biomarkers might have better prognostic values and are attractive to be included in the predictive models, they may not be readily available in limited‐resource settings and are costly to undertake.

In literature, the RISC‐Malawi model has been subjected to external validation in a diverse cohort of hospitalized children from the WHO's study group whose study patients were pooled from 10 studies on pediatric pneumonia from different countries.^44^ In this cohort, there were 17,864 who met the eligibility criteria with a pneumonia case fatality ratio of 4.9%. The RISC‐Malawi score in that validation study had a fair discriminatory value (AUC = 0.75, 95% CI = 0.74−0.77), which was not significantly different from what was obtained in our validation study even though our validation cohort had a higher pneumonia case fatality ratio of 8.7% (Table 2). Furthermore, since calibration statistics were not reported, we could not determine how RISC‐Malawi's calibration measures in the validation study compares with what was obtained in the current study. To our knowledge, Lowlaavar et al. models have not been externally validated in any setting.

### 4.2 Limitations

While the CIN database is a rich source of data routinely collected from several hospitals over a long period and hence suitable for model development and validation, by design, these data were not meant for such purposes. Instead, the CIN was an essential initial step in efforts to understand and improve care in Kenyan hospitals. This led to missing data in variables of interest for many children, resulting in multiply imputing the data, a computationally prohibitive task. However, the CIN dataset had a substantial adequate sample size required for external validation studies.^45^

Lastly, even though we attempted to make the validation population as similar as possible to that used in the derivation of the models we externally validated, we didn't exclude children who carried more than one diagnosis concomitantly, which could explain the reason why the validation case mortality rate is twice to that of the derivation cohort.

### 4.3 Fulfilled knowledge gaps and what to be done next

In the literature, it is more common to find model development studies than validation ones. Hence, many models risk not being utilized in clinical practice because they are yet to be externally validated using a diverse population as expected, thus becoming wasted research efforts. When evaluating a model for risk stratification, researchers should utilize preexisting knowledge and, if available, validate and update an existing model within a similar setting instead of building a new model from scratch with all the drawbacks of overfitting and lack of reproducibility. In this study, we have subjected four models to an external validation study to determine their clinical utility. In the ideal case of perfect validity where scores have AUC ≥ 0.8, calibration intercept = 0, and calibration slope = 1, the model could be recommended for clinical applications. However, if the model deviates from the ideal case, then there is evidence of miscalibration and model recalibration should be performed.^46^, ^47^ Our findings have suggested that the four models have significant miscalibration and hence underscores the necessity of recalibration as a next step. Recalibration is done by adjusting the intercept and slope of the logistic regression equation to match the observed proportion of outcomes in the external dataset. This involves fitting a new logistic regression model with the predicted probabilities from the original model and the observed outcomes in the external dataset. The intercept and slope are then adjusted to align the predicted probabilities with the observed outcomes.

### 4.4 Conclusions

Even though prognostic models underperform when subjected to different populations from the original population used in its derivation, none of the models externally validated in this current study displayed an outstanding discriminatory value of AUC ≥0.8 without under/overestimating the risk based on the calibration statistics. Consequently, these models may not be applied confidently in settings other than those in which they were developed. Based on our findings, recalibrating these models, or further developing prognostic models with greater sensitivity and specificity to identify children at risk of in‐hospital mortality may be warranted.

## AUTHOR CONTRIBUTIONS

**Morris Ogero**: Formal analysis; methodology; visualization; writing—original draft; writing—review and editing. **John Ndiritu**: Methodology. **Rachel Sarguta**: Formal analysis; methodology; writing—original draft; writing—review and editing. **Timothy Tuti**: Formal analysis; methodology; visualization; writing—original draft; writing—review and editing. **Samuel Akech**: Formal analysis; funding acquisition; methodology; validation; visualization; writing—original draft; writing—review and editing.

## CONFLICT OF INTEREST STATEMENT

The authors declare no conflict of interest.

## ETHICS STATEMENT

KEMRI Scientific and Ethical Review Committee approved the CIN study (3459). The Kenya Ministry of Health gave permission for this work which entailed use of deidentified routine patient care data abstracted from medical records after discharge without need for individual patient/clinician consent.

## TRANSPARENCY STATEMENT

The lead author Morris Ogero affirms that this manuscript is an honest, accurate, and transparent account of the study being reported; that no important aspects of the study have been omitted; and that any discrepancies from the study as planned (and, if relevant, registered) have been explained.

## Supporting information

Supporting information.

Supporting information.

## Data Availability

Data for this report are under the primary jurisdiction of the Ministry of Health in Kenya. Enquiries about using the data can be made to the KEMRI‐Wellcome Trust Research Programme Data Governance Committee.

## References

[1] Emi Suzuki . Global Child Mortality Rate Dropped 49% Since 1990. World Bank; 2014.

[2] UNICEF . Levels and trends in child mortality. 2021. https://data.unicef.org/resources/levels-and-trends-in-child-mortality/2022

[3] Ayieko P , Ogero M , Makone B , et al. Characteristics of admissions and variations in the use of basic investigations, treatments and outcomes in Kenyan hospitals within a new Clinical Information Network. Arch Dis Child. 2016;101(3):223‐229. 10.1136/archdischild-2015-309269 26662925 PMC4789757

[4] Sharrow, D , Hug, L , You, D , et al. Global, regional, and national trends in under‐5 mortality between 1990 and 2019 with scenario‐based projections until 2030: a systematic analysis by the UN Inter‐agency Group for Child Mortality Estimation. The Lancet Global Health, 2022;10(2):e195‐e206. 10.1016/s2214-109x(21)00515-5 35063111 PMC8789561

[5] Hands C , Hands S , Verriotis M , et al. Emergency triage assessment and treatment plus (ETAT+): adapting training to strengthen quality improvement and task‐sharing in emergency paediatric care in Sierra Leone. J Glob Health. 2021;11:04069. 34956636 10.7189/jogh.11.04069 PMC8684797

[6] Ayieko P , Ogero M , Makone B , et al. Characteristics of admissions and variations in the use of basic investigations, treatments and outcomes in Kenyan hospitals within a new Clinical Information Network. Arch Dis Child. 2016;101(3):223‐229. 26662925 10.1136/archdischild-2015-309269 PMC4789757

[7] Vogenberg FR . Predictive and prognostic models: implications for healthcare decision‐making in a modern recession. Am Health Drug Benefits. 2009;2(6):218‐222. 25126292 PMC4106488

[8] Ogero M , Sarguta R , Malla L , Aluvaala J , Agweyu A , Akech S . Methodological rigor of prognostic models for predicting in‐hospital paediatric mortality in low‐and middle‐income countries: a systematic review protocol. Wellcome Open Res. 2020;5:106. 32724864 10.12688/wellcomeopenres.15955.1 PMC7364185

[9] Ogero M , Sarguta RJ , Malla L , et al. Prognostic models for predicting in‐hospital paediatric mortality in resource‐limited countries: a systematic review. BMJ Open. 2020;10(10):e035045. 10.1136/bmjopen-2019-035045 PMC7574949 33077558

[10] Feinstein AR . Multivariable Analysis: an Introduction. Yale University Press; 1996:578‐582.

[11] Steyerberg EW , Vergouwe Y . Towards better clinical prediction models: seven steps for development and an ABCD for validation. Eur Heart J. 2014;35(29):1925‐1931. 24898551 10.1093/eurheartj/ehu207 PMC4155437

[12] Collins GS , Reitsma JB , Altman DG , Moons K . Transparent reporting of a multivariable prediction model for individual prognosis or diagnosis (TRIPOD): the TRIPOD statement. BMC Med. 2015;13(1):1. 25563062 10.1186/s12916-014-0241-z PMC4284921

[13] Moons KGM , Altman DG , Reitsma JB , et al. Transparent reporting of a multivariable prediction model for individual prognosis or diagnosis (TRIPOD): explanation and elaboration. Ann Intern Med. 2015;162(1):W1‐W73. 25560730 10.7326/M14-0698

[14] Collins GS , Altman DG . An independent external validation and evaluation of QRISK cardiovascular risk prediction: a prospective open cohort study. BMJ. 2009;339:b2584. 19584409 10.1136/bmj.b2584 PMC2714681

[15] Altman DG , Royston P . What do we mean by validating a prognostic model? Stat Med. 2000;19(4):453‐473. 10694730 10.1002/(sici)1097-0258(20000229)19:4<453::aid-sim350>3.0.co;2-5

[16] Sauerbrei W , Perperoglou A , Schmid M , et al. State of the art in selection of variables and functional forms in multivariable analysis—outstanding issues. Diagnostic and Prognostic Research. 2020;4(1):3. 32266321 10.1186/s41512-020-00074-3 PMC7114804

[17] Altman DG , Vergouwe Y , Royston P , Moons KGM . Prognosis and prognostic research: validating a prognostic model. BMJ. 2009;338:b605. 19477892 10.1136/bmj.b605

[18] Bleeker SE , Moll HA , Steyerberg EW , et al. External validation is necessary in prediction research. JCE. 2003;56(9):826‐832. 14505766 10.1016/s0895-4356(03)00207-5

[19] Olson D , Davis NL , Milazi R , et al. Development of a severity of illness scoring system (inpatient triage, assessment and treatment) for resource‐constrained hospitals in developing countries. Tropical Medicine & International Health: TM & IH. 2013;18(7):871‐878. 10.1111/tmi.12137 23758198 PMC3713504

[20] Reed C , Madhi SA , Klugman KP , et al. Development of the respiratory index of severity in children (RISC) score among young children with respiratory infections in South Africa. PLoS One. 2012;7(1):e27793. 10.1371/journal.pone.0027793 22238570 PMC3251620

[21] George EC , Walker AS , Kiguli S , et al. Predicting mortality in sick African children: the FEAST paediatric emergency triage (PET) score. BMC Med. 2015;13(1):174. 10.1186/s12916-015-0407-3 26228245 PMC4521500

[22] Helbok R , Kendjo E , Issifou S , et al. The Lambaréné organ dysfunction score (LODS) is a simple clinical predictor of fatal malaria in African children. J Infect Dis. 2009;200(12):1834‐1841. 10.1086/648409 19911989

[23] Conroy AL , Hawkes M , Hayford K , et al. Prospective validation of pediatric disease severity scores to predict mortality in Ugandan children presenting with malaria and non‐malaria febrile illness. Crit Care. 2015;19(1):47. 25879892 10.1186/s13054-015-0773-4 PMC4339236

[24] Berkley JA . Prognostic indicators of early and late death in children admitted to district hospital in Kenya: cohort study. BMJ. 2003;326(7385):361. 12586667 10.1136/bmj.326.7385.361 PMC148891

[25] Hooli S , Colbourn T , Lufesi N , et al. Predicting hospitalised paediatric pneumonia mortality risk: an external validation of RISC and mRISC, and local tool development (RISC‐Malawi) from Malawi. PLoS One. 2016;11(12):e0168126. 10.1371/journal.pone.0168126 28030608 PMC5193399

[26] Lowlaavar N , Larson CP , Kumbakumba E , et al. Pediatric in‐hospital death from infectious disease in Uganda: derivation of clinical prediction models. PLoS One. 2016;11(3):e0150683. 26963914 10.1371/journal.pone.0150683 PMC4786260

[27] Ministry of Health (MOH) [Kenya] . Paediatric admitting record form. Kenya: Ministry of Health; 2015. http://www.idoc-africa.org/images/documents/Paeds%202_a_-%20PAR%20Paediatric%20Admitting%20Record%20Form.pdf2016

[28] Harris PA , Taylor R , Thielke R , Payne J , Gonzalez N , Conde JG . Research electronic data capture (REDCap)—a metadata‐driven methodology and workflow process for providing translational research informatics support. J Biomed Inf. 2009;42(2):377‐381. 10.1016/j.jbi.2008.08.010 PMC2700030 18929686

[29] Irimu G , Ogero M , Mbevi G , et al. Tackling health professionals' strikes: an essential part of health system strengthening in Kenya. BMJ Glob Health. 2018;3(6):e001136. 10.1136/bmjgh-2018-001136 PMC6278918 30588346

[30] World Health Organization . Boys weight for age z‐score, 2021.

[31] World Health Organization . Girls weight for age z‐score, 2021.

[32] Centers for Disease Control and Prevention . National Center for Health Statistics. In: Files Z‐sD, ed., 2021.

[33] Akech S , Chepkirui M , Ogero M , et al. The clinical profile of severe pediatric malaria in an area targeted for routine RTS, S/AS01 malaria vaccination in Western Kenya. Clin Infect Dis. 2020;71(2):372‐380. 31504308 10.1093/cid/ciz844 PMC7353324

[34] Bijlsma MW , Brouwer MC , Bossuyt PM , et al. Risk scores for outcome in bacterial meningitis: systematic review and external validation study. J Infect. 2016;73(5):393‐401. 27519619 10.1016/j.jinf.2016.08.003

[35] Muller MP , Tomlinson G , Marrie TJ , et al. Can routine laboratory tests discriminate between severe acute respiratory syndrome and other causes of community‐acquired pneumonia? Clin Infect Dis. 2005;40(8):1079‐1086. 15791504 10.1086/428577 PMC7107805

[36] Van Calster B , McLernon DJ , Van Smeden M , Wynants L , Steyerberg EW . Calibration: the Achilles heel of predictive analytics. BMC Med. 2019;17(1):230. 31842878 10.1186/s12916-019-1466-7 PMC6912996

[37] CalibrationCurves: Calibration performance [program] . R package version 0.1.2 version, 2016.

[38] Sterne JAC , White IR , Carlin JB , et al. Multiple imputation for missing data in epidemiological and clinical research: potential and pitfalls. BMJ. 2009;338:b2393. 19564179 10.1136/bmj.b2393 PMC2714692

[39] White IR , Royston P , Wood AM . Multiple imputation using chained equations: issues and guidance for practice. Stat Med. 2011;30(4):377‐399. 10.1002/sim.4067 21225900

[40] Rubin DB . Multiple Imputation for Nonresponse in Surveys. John Wiley & Sons; 2004.

[41] Buuren S , Groothuis‐Oudshoorn K . Mice: multivariate imputation by chained equations in R. J Stat Softw. 2011;45(3):1548‐7660.

[42] Florin TA , Ambroggio L , Brokamp C , et al. Reliability of examination findings in suspected community‐acquired pneumonia. Pediatrics. 2017;140(3):e20170310. 28835381 10.1542/peds.2017-0310 PMC5574720

[43] Sjoding MW , Cooke CR , Iwashyna TJ , Hofer TP . Acute respiratory distress syndrome measurement error. Potential effect on clinical study results. Ann Am Thorac Soc. 2016;13(7):1123‐1128. 27159648 10.1513/AnnalsATS.201601-072OC PMC5015753

[44] Rees CA , Hooli S , King C , et al. External validation of the RISC, RISC‐Malawi, and PERCH clinical prediction rules to identify risk of death in children hospitalized with pneumonia. J Glob Health. 2021;11:04062. 34737862 10.7189/jogh.11.04062 PMC8542381

[45] Vergouwe Y , Steyerberg EW , Eijkemans MJC , Habbema JDF . Substantial effective sample sizes were required for external validation studies of predictive logistic regression models. JCE. 2005;58(5):475‐483. 15845334 10.1016/j.jclinepi.2004.06.017

[46] Steyerberg EW , Borsboom GJJM , van Houwelingen HC , Eijkemans MJC , Habbema JDF . Validation and updating of predictive logistic regression models: a study on sample size and shrinkage. Stat Med. 2004;23(16):2567‐2586. 15287085 10.1002/sim.1844

[47] Steyerberg EW , Harrell Jr. FE , Borsboom GJJM , Eijkemans MJC , Vergouwe Y , Habbema JDF . Internal validation of predictive models. JCE. 2001;54(8):774‐781. 11470385 10.1016/s0895-4356(01)00341-9

