# Supplementary material for: Pediatric prognostic models predicting inhospital child mortality in resource‐limited settings: An external validation study
Source: Health Sci Rep. 2023 Aug 27;6(8):e1433. doi: 10.1002/hsr2.1433 (PMC10460931; doi:10.1002/hsr2.1433)
Supplement: Supplementary file 1 — Supporting information. [file HSR2-6-e1433-s002.pdf]

Paediatric Admission Record – Paediatric Ward

**Name**		**IP No.**		**Ward**	

Contact (Tel)		Relation		DOB	dd/mm/yyyy
Admission Date	dd /mm / yyyy	Sex	M ☐ / F☐	Age	years months days
Subcounty/ Constituency		Location		Sub-location	
Village		Nearest Health Facility		Nearest School	
Is this first admission since birth?	Y☐ N☐	No of previous admissions		Date of last admission	dd/mm/yyyy
Re-admission to this hospital?	Y☐ N☐	Discharged <1 month ago	Y☐ N ☐		
Is child referred from another health facility?	Y☐ N☐	Date first treated in other facility	dd/mm/yyyy		

**Presenting Complaints**	

History

**Weight**	Kg	**Height / Length**	cm	*WHZ score*		MUAC (cm)		**Head Circum** (cm)	

Length of illness	days	
Fever – No. of days =	**Y**☐	**N**☐
Cough– No. of days =	**Y**☐	**N**☐
Cough > 2 weeks	**Y**☐	**N**☐
Contact with TB /Chronic cough (last 12 months)	**Y**☐	**N**☐
Difficulty breathing	**Y**☐	**N**☐
Diarrhoea No. of days =	**Y**☐	**N**☐
Diarrhoea > 14d	**Y**☐	**N**☐
Diarrhoea bloody	**Y**☐	**N**☐
Vomiting, No / 24hrs =	**Y**☐	**N**☐
Vomits everything	**Y**☐	**N**☐
Difficulty feeding	**Y**☐	**N**☐
Convulsions Number in last 24hrs =_____	**Y**☐	**N**☐
Partial / focal fits?	**Y**☐	**N**☐
Passing blood/tea/cola coloured urine?	**Y**☐	**N**☐
Sleeps under mosquito net	**Y**☐	**N**☐

Pre-existing illness	None ☐
Drugs taken last 2 weeks	None ☐
Any vaccine reaction suspected?	**Y** ☐ N☐<br>If yes, indicate most recent vaccine:

Additional history of presenting illness;

Immunization

Vaccination card available? **Y**☐ **N**☐

Has child received any vaccinations since birth? **Y** ☐ N☐

Vaccine	Received	Date received
BCG	**Y**☐ **N**☐ Don't Know ☐	dd /mm / yyyy
OPV 0(Birth)	**Y**☐ **N**☐ Don't Know ☐	dd /mm / yyyy
OPV/Penta/PCV 1	**Y**☐ **N**☐ Don't Know ☐	dd /mm / yyyy
Rota 1	**Y**☐ **N**☐ Don't Know ☐	dd /mm / yyyy
OPV/Penta/PCV 2	**Y**☐ **N**☐ Don't Know ☐	dd /mm / yyyy
Rota 2	**Y**☐ **N**☐ Don't Know ☐	dd /mm / yyyy
OPV/Penta/PCV 3	**Y**☐ **N**☐ Don't Know ☐	dd /mm / yyyy
IPV	**Y**☐ **N**☐ Don't Know ☐	dd /mm / yyyy
RTS,S 1(Malaria)	**Y**☐ **N**☐ Don't Know ☐	dd /mm / yyyy
RTS,S 2(Malaria)	**Y**☐ **N**☐ Don't Know ☐	dd /mm / yyyy
RTS,S 3(Malaria)	**Y**☐ **N**☐ Don't Know ☐	dd /mm / yyyy
RTS,S 4(Malaria)	**Y**☐ **N**☐ Don't Know ☐	dd /mm / yyyy
Measles 1	**Y**☐ **N**☐ Don't Know ☐	dd /mm / yyyy
Measles 2/MR	**Y**☐ **N**☐ Don't Know ☐	dd /mm / yyyy

Vitamin A given within last 6months? **Y**☐ **N**☐

Birth/Antenatal History

Maternal PMTCT status: ☐ Positive ☐ Negative ☐ Unknown

Growth and Development:

Family/Social history:

Nutritional history:

Review of Systems:

Respiratory including ENT

Cardiovascular

Gastro-intestinal / Genitourinary

Examination

Vital Signs	Temp	^{0}C	Resp Rate	bpm	HR	/min	O2 Sat	%	BP	mmHg

General Examination

Oral thrush **Y**☐ **N**☐ Lymph N > 1cm **Y**☐ **N**☐

Finger Clubbing Y☐ N☐

Eye signs of malnutrition?

Pus☐ ulceration☐ None☐

Jaundice	**Y**☐	**N**☐

Oedema (tick all that apply)	☐None ☐Foot ☐Knee ☐Face

**A**	Stridor	**Y**☐	**N**☐
**B**	Central Cyanosis	**Y**☐	**N**☐
	Indrawing	**Y**☐	**N**☐
	Grunting	**Y**☐	**N**☐
	Acidotic breathing	**Y**☐	**N**☐
	Wheeze	**Y**☐	**N**☐
	Crackles	**Y**☐	**N**☐
**Circ & Dehy dr'n**	Peripheral Pulse	☐Normal	☐ Weak
	Cap Refill	secs	*X = not possible*
	Skin warm at:	☐ Hand ☐ Elbow ☐Shoulder	
	Pallor / Anaemia	0☐ +☐ +++☐	
	Sunken eyes	**Y**☐	**N**☐
	Skin pinch (sec)	**0** **1**	**≥ 2**
**D**	**AVPU**	**A** **V** **P**	**U**
	Can drink / breastfeed?	**Y**☐	**N**☐
	Stiff neck	**Y**☐	**N**☐
	Bulging fontanelle	**Y**☐	**N**☐
	Can sit without support during this illness	**Y**☐	**N**☐
Infant < 1yr	Irritable	**Y**☐	**N**☐
	Reduced movement / tone	**Y**☐	**N**☐
	Umbilicus	Normal ☐ Pus ☐	Pus & red skin ☐

Abdomen

Rt Lt

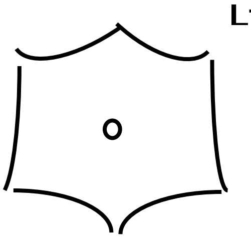

Chest

R L

Front

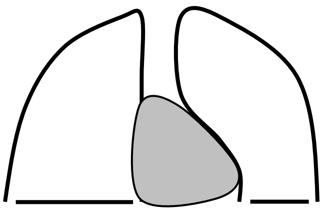

Back

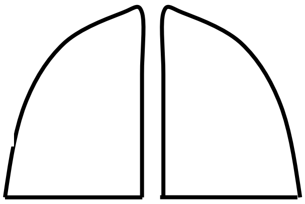

CVS

Blantyre Coma Score=_____

Motor response	Verbal response	Eye response
☐Localises pain=2	☐ Normal response=2	☐ Following objects=1
☐Withdraws to pain =1	☐ Inappropriate=1	☐ Not following =0
☐No response =0	☐ No response=0	

Posture (tick that apply)

Normal	Opisthotonus	Decerebrate	Decorticate

Blood/tea/cola coloured urine observed?

Y☐ N ☐ Urine not seen ☐

Bones & Joints

Wrist / Rib signs Rickets **Y**☐ **N**☐

**ENT exam**	Neurological Examination (normal, increased↑, reduced↓)
Rt Ear<br>Lt Ear<br>Nose<br>Throat	

	Normal	Right side		Left side	
		Upper limb	Lower limb	Upper limb	Lower limb
**Power:**					
**Tone:**					
**Reflexes:**					
**Plantar responses:**					
**Senstation:**					

Summary of presentation & problems

……
……
……
……

Investigations Ordered (record subsequent tests and all results in medical record)

Malaria	☐ Blood slide ☐ Rapid Test	***Glucose***	☐ Stick test ☐ Laboratory
Haematology	☐ Hb ☐ Full haemogram ☐ PCV	***Chemistry***	☐ Na/K ☐ U&C ☐ Ca+Phos ☐ Alb ☐ LFT
Microbiology	☐ Lumbar Puncture ☐ Blood Culture	***HIV***	☐ Rapid test ☐ PCR
X-Ray	☐ CXR ☐Wrist Other =	***Urine***	☐ Urinalysis ☐ Micro & culture
TB Test	☐ Microscopy for AAFBs ☐Mantoux<br>☐ Xpert MTB/RIF ☐ *Myco.TB* culture	***Stool***	☐ Microscopy ☐ Micro & culture
		Other	

Admission Diagnoses – Select ONE *primary diagnosis (tick box indicating "1") and ANY secondary diagnoses (tick box indicating "2"), then indicate level of severity or type of disease if required*

Malaria	1 ☐ 2 ☐	☐ Severe ☐ Non-severe	***Anaemia***	1 ☐ 2 ☐	☐ Severe ☐ Non-severe
Pneumonia	1 ☐ 2 ☐	☐ Severe ☐ Non-severe	***Sickle cell disease***	**1** ☐ **2** ☐	
Diarrhoea	1 ☐ 2 ☐	☐ Non-bloody ☐ Bloody (dysentery)	***Meningitis***	**1** ☐ **2** ☐	
Dehydration	1 ☐ 2 ☐	☐ Shock ☐ Severe ☐ Some ☐ None	***Rickets***	**1** ☐ **2** ☐	
HIV result		☐ Positive ☐ Exposed /PMTCT +<br>☐ Negative ☐ Declined test<br>☐On HAART ☐ indeterminate	***Asthma***	**1**☐ **2**☐	☐ Severe<br>☐ Mild/moderate
Malnutrition	1 ☐ 2 ☐	☐ Kwash ☐ Marasm ☐ M. Kwash<br>☐ Moderate malnutrition ☐mild/none	***Suspected TB***	**1** ☐ **2** ☐	
			Prematurity / LBW	**1** ☐ **2** ☐	
Other 1	1 ☐ 2 ☐		***Neonatal sepsis***	**1** ☐ **2** ☐	
Other 2	1 ☐ 2 ☐				

Clinician Name & Sign

………….. ………….. Date… /……/…… Time ....
dd/mm/yyyy am / pm
