# Supplementary material for: Pediatric prognostic models predicting inhospital child mortality in resource‐limited settings: An external validation study
Source: Health Sci Rep. 2023 Aug 27;6(8):e1433. doi: 10.1002/hsr2.1433 (PMC10460931; doi:10.1002/hsr2.1433)
Supplement: Supplementary file 2 — Supporting information. [file HSR2-6-e1433-s001.docx]

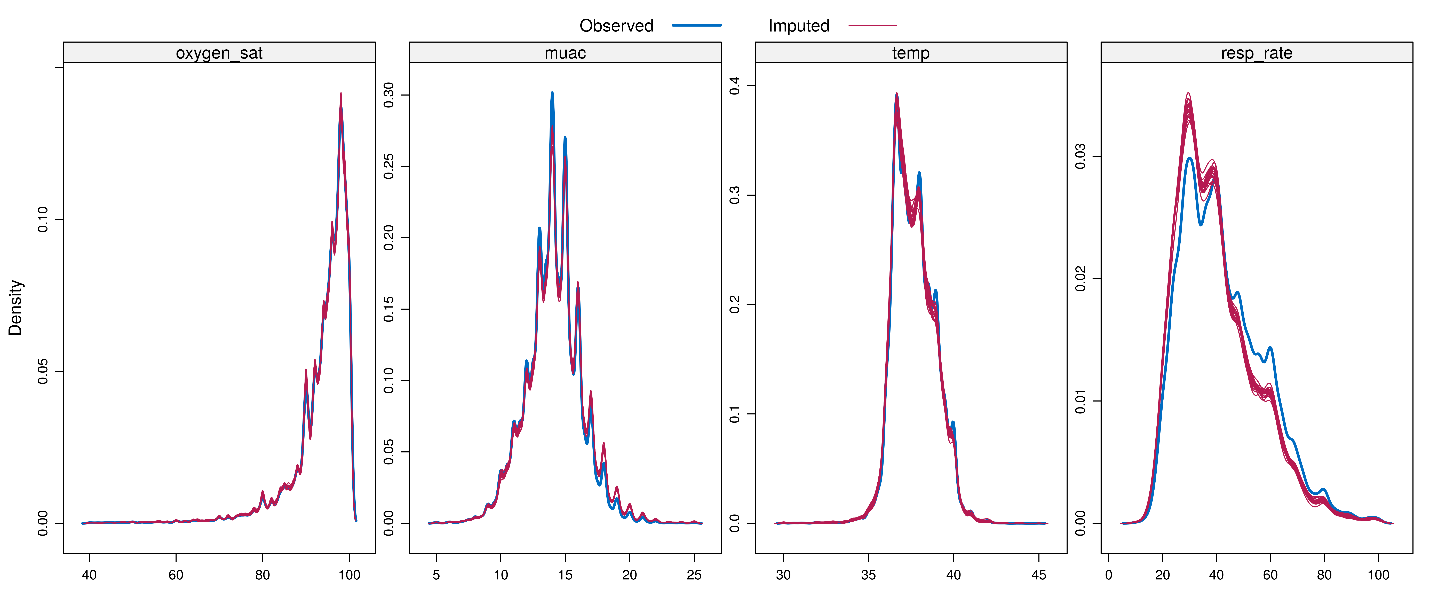


**Supplementary figure 1: Density functions of the observed and imputed numerical variables with high data missingness.**


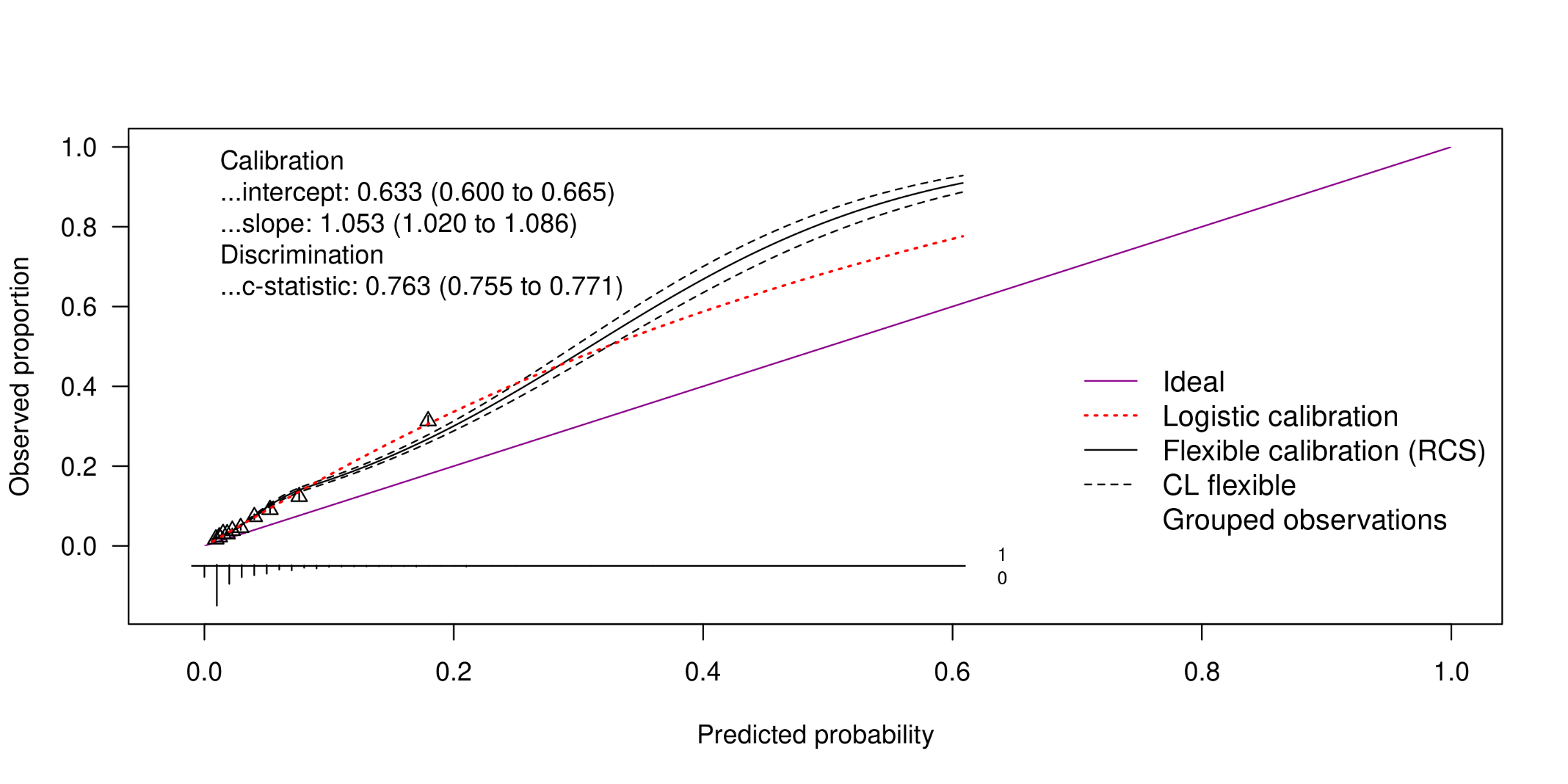


**Supplementary figure 2: Performance of the RISC-Malawi model in a sensitivity analyses dataset (Pneumonia is defined based on the admission clinical diagnosis instead of danger signs). The values show calibration curves and other model performance metrics. Key: RCS denotes the Restricted Cubic Splines, and CL denotes the Confidence Limits (95%).**


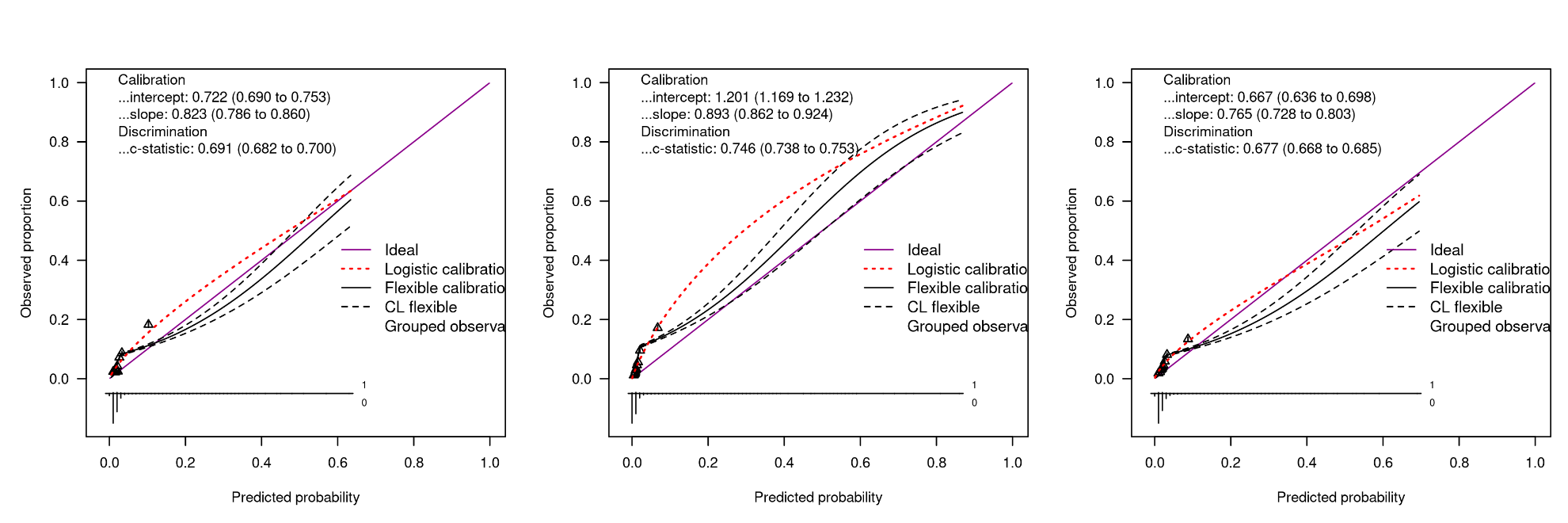
**Supplementary figure 3: Performance of the Lowlaavar *et al* 2016 models in an external validation dataset whereby Abnormal Blantyre coma score was defined using the disability scale of AVPU (Alert, Verbal response, Pain response, Unresponsive) such that patients who were not alert but responding to verbal stimuli were assumed to have abnormal Blantyre coma score. The first panel to the left is the calibration curves of the primary model (Model I), the panel in middle are the calibration curves for model II, and the last panel to the right are the calibration curves of the model III. Key: RCS denotes the Restricted Cubic Splines, and CL denotes the Confidence Limits (95%).**
